# Optical tissue clearing associated with 3D imaging: application in preclinical and clinical studies

**DOI:** 10.1007/s00418-022-02081-5

**Published:** 2022-03-02

**Authors:** Cinzia Brenna, Carolina Simioni, Gabriele Varano, Ilaria Conti, Eva Costanzi, Mattia Melloni, Luca Maria Neri

**Affiliations:** 1 Department of Translational Medicine, University of Ferrara, 44121 Ferrara, Italy; 2 Medical Research Center, Medical Faculty Mannheim, University of Heidelberg, Theodor-Kutzer-Ufer 1-3, 68167 Mannheim, Germany; 3 Department of Life Sciences and Biotechnology, University of Ferrara, 44121 Ferrara, Italy; 4 LTTA – Electron Microscopy Center, University of Ferrara, 44121 Ferrara, Italy

**Keywords:** OTC, 3D imaging, Clinical diagnosis, Clinical applications, Oncology

## Abstract

Understanding the inner morphology of intact tissues is one of the most competitive challenges in modern biology. Since the beginning of the twentieth century, optical tissue clearing (OTC) has provided solutions for volumetric imaging, allowing the microscopic visualization of thick sections of tissue, organoids, up to whole organs and organisms (for example, mouse or rat). Recently, tissue clearing has also been introduced in clinical settings to achieve a more accurate diagnosis with the support of 3D imaging. This review aims to give an overview of the most recent developments in OTC and 3D imaging and to illustrate their role in the field of medical diagnosis, with a specific focus on clinical applications.

**Graphical abstract:**

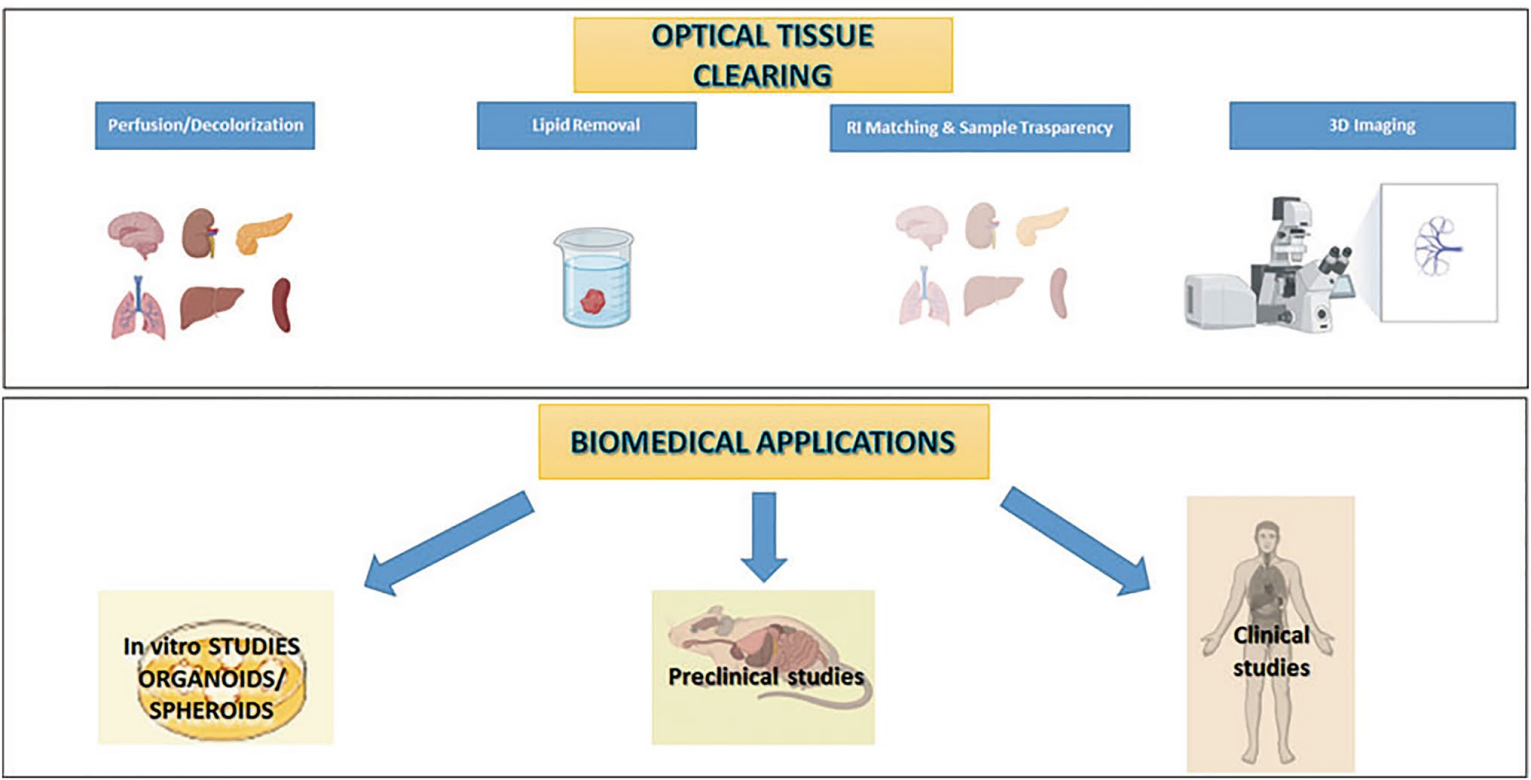

## Introduction

Optical tissue clearing (OTC) defines a wide set of protocols, aiming to make large and fixed biological samples optically transparent, where “large” refers to thick sections of tissue, organs, up to entire organisms (for example, mouse or rat) (Ariel 2017). The principle it is based on is the removal of the major sources of light scattering into the tissue, as, for instance, lipids and aqueous media interfaces, which generally create a mismatch of the refractive index and the consequent “milky appearance” that does not permit the light to propagate in straight lines (that is, the light scattering phenomenon) (Christopher 2006; Genina 2010; Richardson and Lichtman 2015).

**Fig. 1:**
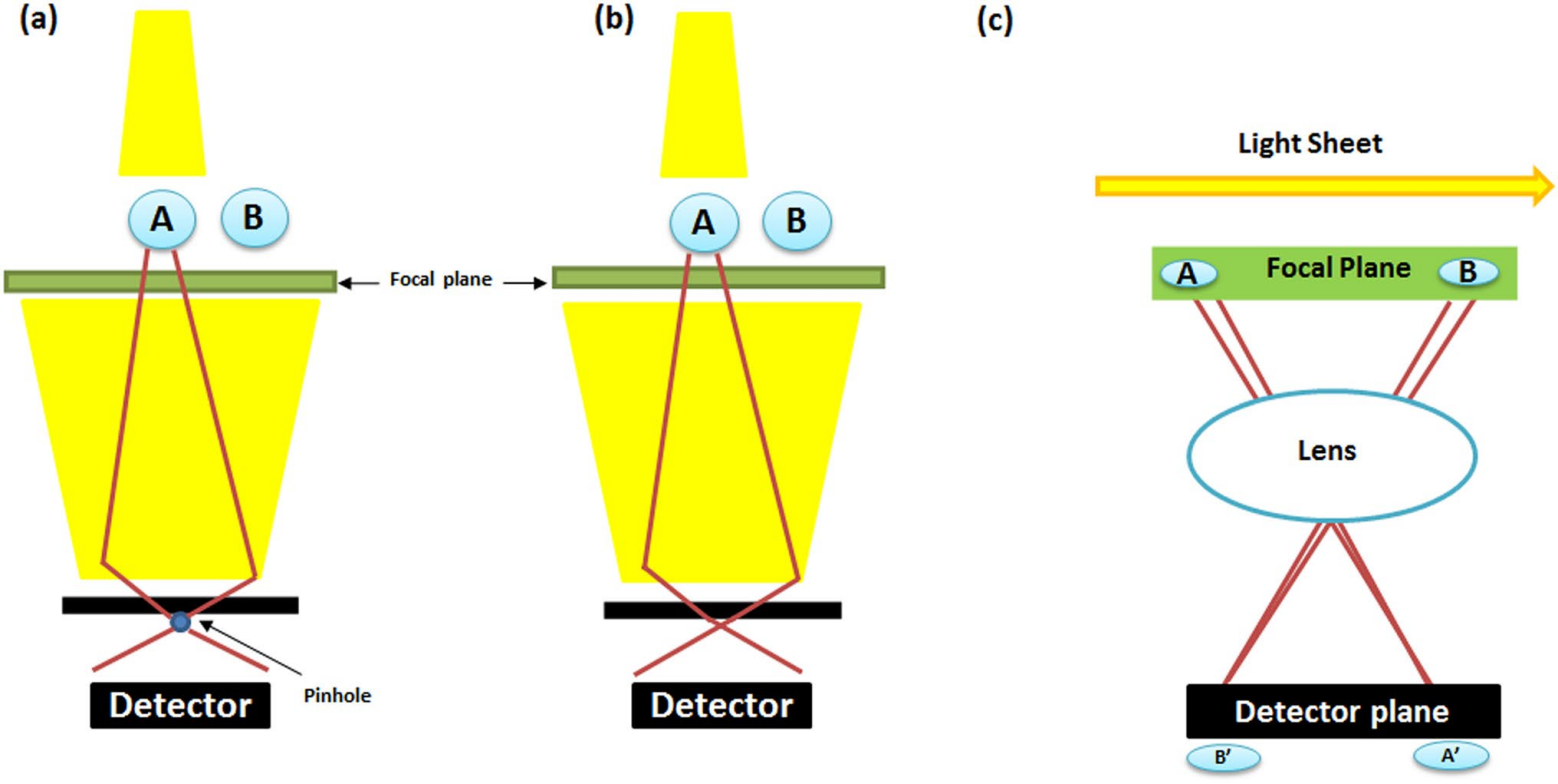
Schematic workflow of the mode of action of confocal (**a**), two photon (**b**), and light sheet (**c**) microscopies. In diagrams (**a**) and (**b**), point A is represented in the focal plane, whereas point B is outside. Only the light sheet microscopy creates a light plane coincident with the focal plane, allowing the focus localization inside both of the points: https://biorender.com/

In recent decades, the increasing need for a deeper understanding of morphological and developmental processes within organisms required more precise methodologies for three-dimensional (3D) imaging (Richardson and Lichtman 2015), using, for instance, confocal two-photon and light-sheet microscopy (Minsky, 1955 U.S. patent n° 3013467; Minsky 1988; Denk 1990; Keller and Dodt 2012). A schematic illustration of the operating principle of these microscopes is reported in Fig. 1.

The importance of 3D imaging lies in solving the drawbacks of two-dimensional (2D) microscopic analysis (i.e., the time-consuming and prone-to-error sample preparation, the tissue degradation—due to the massive use of reagents—and slicing, resulting in reduced thickness and a consequent limited spatial resolution) (van Royen 2016; Poola 2019). The 3D imaging can undoubtedly improve the visualization of the samples, resulting in a higher amount of structural information provided (which is very small, considering the thickness of the 2D slices), as well as the spatial resolution (highly limited in the 2D) (Richardson and Lichtman 2015; Seo 2016). Moreover, when using 2D thin slices, the sample 3D reconstruction becomes hard to perform (Gómez-Gaviro 2020). Although some image-processing software might create histological 3D reconstructions, such as Fiji (Schindelin 2012), MATLAB (Křížek 2016), or Imaris (Au-Haass-Koffler 2012), this image stacking process is laborious, time-consuming, prone to error, and not wholly reliable (Gómez-Gaviro 2020).

On the other hand, a nontransparent tissue is not suitable for acquiring 3D high-resolution images due to the light scattering phenomenon. Therefore, so far, the best compromise to obtain 3D high-resolution images is to render them optically transparent by using clearing reagents (Gómez-Gaviro 2020).

To date, OTC has been extensively used for the investigation of the structural architecture of other organs, especially kidney, muscle, bone, dental tissue, and human placenta, where the morphology of placental blood barriers was also evaluated (Winkler 2010; Kay 2013; Pauli 2017; Klingberg 2017; Carrillo 2018; Jing 2019; Huang 2019; Williams 2019). As demonstrated by Cai and coworkers (2019), who imaged a whole murine organism, OTC represents an essential tool that can be applied to a wide range of specimens (that is, a variety of organs from rodents, primates to humans), in a fast, safe, economical, easy-to-use, and highly reproducible way, at a single-cell resolution (Silvestri 2016; Kubota 2017). Actually, there is no universal OTC protocol, but rather the choice of one method depends on the sample, the specimens, and the fluorescent marker used. Kolesová and coworkers (2016) gave a clear example of this, testing and comparing five different OTC protocols [tetrahydrofuran dehydration and dibenzylether protocol (THF-DBE), SCALE, CLARITY, and CUBIC], and three imaging methods intended to preserve Green fluorescent protein (GFP) fluorescence in the central nervous system and for evaluating their ability to make mouse hearts and whole embryos transparent, concluding that the optimal method depends on the detail level required (Kolesová 2016).

Recently, it has been proposed to apply OTC approaches to discriminate between healthy and pathologic tissues to improve clinical and diagnostic settings, until now circumscribed only to 2D imaging. For instance, different human cleared biopsies were analyzed by 3D imaging (breast, prostate, kidney, brain, gingiva, skin, and temporal bones; Glaser 2017; Poola 2019). Moreover, OTC feasibility in clinical applications has been demonstrated by ascertaining histological differences between human benign and tumor prostate, as well as pancreatic lesions and adenocarcinomas (Hong 2019; Matryba 2019; van Royen 2016).

This innovative OTC-3D approach led to two great advantages: whole-mount tissue instead of thin slices and the acquisition of images at cellular resolution, which is essential, considering that the early stages of several pathologies depend on changes in the cellular morphology and functions (Antonacci and Braakman 2016).

The novelty of OTC-3D medical application imaging is the use of entire biopsies marked by specific fluorescent probes, making it a cost-effective, nonradioactive mode of imaging for cancer detection (Martinelli 2020).

Recent findings in OTC-3D imaging are focused on the improvement of near-infrared probes. This spectrum range (700–850 nm) permits absorption of light deeper than others emitting at 400–600 nm, allowing a better tissue visualization and resulting in lower light absorption and minor autofluorescence by blood and other components in this spectrum range (Martinelli 2020). Moreover, cancer tissues are often characterized by necrotic areas, which might not be imaged in standard conditions. Generally speaking, clearing agents combined with near-infrared probes enhance the light penetration in biological samples. In addition, lipid and natural pigment removal increase the refractive index matching and the spatial resolution, allowing a better sample analysis (Gómez-Gaviro 2020).

This review explores how OTC-3D imaging could be used in preclinical and clinical cancer research and other possible methods of life imaging and 3D visualization (not necessarily needing tissue clearing) as alternative approaches, currently used in the diagnostic field.

## 3D in vitro cancer research: the use of cleared organoids and spheroids

Organoids are defined as human pluripotent or organ restricted (i.e., stomach, liver, or bladder) stem cell-derived 3D structures, which mimic cell heterogeneity, native histologic architectures, and cell–extracellular matrix interactions (i.e., breast, colon, pancreas, prostate, ovary—Eiraku 2008; Ham 2016; Ham 2017; Colella 2018; Khawar 2018; Lazzari 2018). Due to their self-assembling ability, organoids can grow into microscopic versions of parent organs suitable for 3D study (Perkhofer 2018; Dekkers 2019). As new 3D cellular models, organoids have been established and massively used in biology, oncology, and pharmacology (Lancaster 2013; Alépée 2014), representing an innovative tool for personalized and cancer medicine (Perkhofer 2018). Cancer is a multifactorial process based on genetic and environmental factors, and, currently, the study approaches are based above all on cultured tumor cell lines and animal models.

Compared with 2D and animal models, as well as with the whole-mount 3D imaging, the benefits in using organoids correlate in the following points: (a) 2D in vivo cellular models exhibit some downsides. Cells do not correctly interact between each other or with the surrounding extracellular matrix, especially in plastic surfaces, where the presence of oxygen and nutrients can be limited, further weakening the possibility to perform more accurate and precise cellular studies (Zschenker 2012; Duval 2017; Nürnberg 2020). Moreover, tumor cell lines may accumulate further mutations due to environmental interactions not representing the human complexity; (b) the genetic background of animal cancer models does not completely reflect human physiology (Perkhofer 2018). Therefore, human organoids may be considered a novel experimental model that overcomes the gap between animal models and human trials (Kim 2020); (c) the possibility to directly study human material, especially considering the accessibility of tumor material from patients (Perkhofer 2018); and (d) the limitations that are given by using only biopsies.

On the other hand, the use of organoids is often debated. The reasons could be the following: (a) organoids exhibit a superior morphology for glandular tissues but not appropriate for stratified tissue, as skin; (b) the organoid cultures were established to investigate normal cellular differentiation in the prostate (Lang 2001) and breast (Barcellos-Hoff 1989). In addition, it is not clear how tumor cells interact with basement membrane gel; (c) in vivo, tumor growth is anchorage-independent, but organoids are anchorage-dependent, given their adherence to the basement membrane proteins in the gels; (d) in addition, in vivo, tumor cells grow as solid masses, whereas in the organoid system, they grow as hollow spheres, not recreating the actual patient morphology, so they do not always recreate patient tissue architecture (Lang 2019); (e) tumor tissues can also be contaminated by normal cells: a study of in vitro and in vivo models performed on tumor organoids showed a high percentage of normal cells (Pauli 2017). Therefore, when using organoids, it is fundamental to confirm the absence of all potential contaminants (Lang 2019); and (f) heterogeneity of organoids, which could be critical for discerning the ratio and type of cells (tumor, normal, different tumor clones).

Nevertheless, fluorescence microscopy, coupled with OTC and 3D imaging, has extensively contributed to characterizing organoid cellular composition and demonstrating their resemblance to the original tissue (Dekkers 2019). Furthermore, different OTC protocols on human cell-derived organoids have been established (Cora 2019; Dekkers 2019; Krieger 2020), allowing the study of the structural complexity, the map of the spatial distribution, phenotypic identity, and cellular state of all individual cells composing these 3D structures (Costa 2019; van Ineveld 2020). The principal advantages of these new approaches regard the suitability for different species and a wide variety of organs and the reduced processing time if compared with the classic OTC procedures, such as BABB, CLARITY, and others. A recent work published by Krieger et al. (2020) exemplarily demonstrated a similar approach in glioblastoma. This research showed that tumor cells within organoids mimic the in vivo glioblastoma multiforme (GBM) behavior in progression and invasiveness. Moreover, the transcriptional changes implicated in the invasion process have been reported and proved coherent with the GBM cell behavior, indicating that GBM cells could reactively upregulate genes required for their dispersion (Krieger 2020).

Compared to the conventional 2D methodology, the use of OTC-3D imaging to organoids allows deciphering complex cell morphology and tissue architecture. Furthermore, compared with the first OTCs, other approaches have already implemented a second generation of protocols, which is more undemanding and easy to enhance in laboratories. For example, the system designed by Rios and coworkers (2019) and shortened by van Ineveld and coworkers in a 3-day protocol, based on the use of a new clearing agent named FUnGI (fructose, urea, and glycerol for imaging), provided an organoid-clearing protocol for multi-color lineage tracing of tumor heterogeneity, which proved suitable both for intact organoids from different species, and organs (Rios 2019; van Ineveld 2020).

The 3D imaging of cleared organoids is compatible with different samples, rendering this approach the new frontier for studying different diseases and cancers and laying the foundations for a more precise personalized medicine.

## Preclinical and clinical applications of optical tissue clearing

To date, conventional 2D histology is still the method of choice, although prone to limitations, in clinical and, in particular, in cancer research (Feuchtinger 2016). 2D histology is considered a reductionist approach, as the analysis of a single 2D tissue slice provides information about only a small percentage of the entire tumor area that will be detectable for studying, with a very high risk of false positives (Feuchtinger 2016; Almagro 2021). In the tumor analysis process, the definition of its growth rate from complex morphological structures is relevant, such as tumor vasculature, that can only be recognized and fully understood in its three dimensions. OTC-3D provides panoramic visualization of cancer-bearing organs and constitutes a powerful tool to analyze the tumor architecture, microenvironment, heterogeneity, and progression both in preclinical models and in patients (Almagro 2021). Various tissue-clearing methods (aqueous-, organic-, or hydrogel-based) were tested on tumor areas in preclinical models that revealed different degrees of optical transparency, tissue preservation, fluorescent signal conservation, and compatibility with 3D imaging and standard confocal microscopy (Lloyd-Lewis 2016). Table 1 provides an overview of the state of the art of OTC and their preclinical and clinical applications. Listing all the OTCs is beyond the scope of this table; instead, we want to focus on the more successful protocols used in preclinical and clinical models. The full name of the OTCs mentioned in the table is listed in the section “Abbreviations.”

**Table 1:** Overview of preclinical and clinical application of the most used optical clearing methods

	Protocol name	Chemicals used	Preclinical application	Clinical application
Simple immersion	Formamide	Formamide	Data not reported	Data not reported
	FRUIT	Fructose/thioglycerol/urea	Data not reported	Data not reported
	PEGASOS	Polyethylene glycol (PEG)-associated solvent system	Data not reported	Data not reported
	SeeDB series	Fructose/thioglycerol	Davis (2016)	Data not reported
	Sucrose	Sucrose	Data not reported	Data not reported
Solvent-based	BABB (series)	Benzoic acid/benzyl benzoate	Lang et al. (2001); Dobosz, et al. (2014)	Data not reported
	DISCO (series)	Dichlormethane/DBE	Ertürk et al. (2012); Tainaka et al. (2014); Hong et al. (2020)	Tainaka (2014); Hong (2020)
	ECi (series)	Ethyl-3-phenylprop-2-enoate	van Royen et al. (2016); Brenna et al. (2020)	(Avilov 2021)
	FUnGI	Fructose, urea, glycerol	Roi et al. (2019); van Ineveld et al. (2020); Dawson et al. (2021)	Data not reported
	THF-DBE	Tetrahydrofuran dehydration and dibenzylether	Ertürk et al. (2012); Wang et al. (2018); Dawson et al. (2021)	Data not reported
Hyperhydratation	CUBIC	4 M Urea/50% sucrose	Davis et al. (2016); Guldner et al. (2016); Kolesová et al. (2016) Lloyd-Lewis et al. (2016); Kubota et al. (2017); Tainaka et al. (2018)	Nojima (2017)
	Scale (series)	4 M Urea/sorbitol	Kolesová et al. 2016	Data not reported
Hydrogel embedding	ACT-PRESTO	Hydrogel/sodium dodecyl sulfate (SDS)	Data not reported	Data not reported
	CLARITY	FocusClear/80% glycerol	Kolesová et al. (2016), Lagerweij et al. (2017)	Glaser (2017), Hsueh (2017), Chen (2019)
	ExM	Hydrogel	Brenna et al. (2020), Sun et al. (2020)	Sun (2020)
	MyoClear	Hydrogel monomer solution	Williams et al. (2019)	Data not reported
	PACT	Hydrogel/SDS	Guldner et al. (2016)	Data not reported

### Preclinical approaches of OTC

Aqueous methodologies (i.e., fructose, glycerol, Scale, and SeeDB series) are easy to handle and have low toxicity. In addition, there is no significant volume change, and the fluorescence retainment is considerably higher than in organic protocols.

Generally speaking, most of the aqueous protocols do not include delipidation, causing a low rate of transparency/translucency for whole mouse organs in healthy and diseased conditions (Lang 2001; Dobosz 2014; Cai 2018).

The second-generation aqueous OTCs were optimized to overcome this problem. This is the case with SeeDB, which, in the beginning, was conceived for studying the brain’s connectome. Afterward, its use was extended to other organs, both in healthy and pathologic conditions. For enhancing the visualization of mammary ductal and lobulo-alveolar structures, SeeDB and CUBIC protocols were considered superior for volumetric fluorescence imaging and whole-mount histochemical staining, respectively (Lloyd-Lewis 2016).

### Solvent-based OTCs in preclinical models

CUBIC is often considered the method of choice. The main advantage is that CUBIC chemicals elute endogenous chromophores (e.g., hemoglobin), reducing blood autofluorescence (Susaki 2014; Tainaka 2014; Kolesová 2016, 2021). In addition, CUBIC preserves fluorescence, allowing the protocol’s performance after the immunolabeling, especially regarding whole-mount GFP cardiac mouse samples (Susaki 2014; Tainaka 2014; Kolesová 2016, 2021).

CUBIC protocol was optimized to optically clear mouse bones and detect cancer metastasis (e.g., lung, kidney) in different mouse models, for instance, by imaging the triple-positive breast cancer metastasis in bones and brain (Kubota 2017; Tainaka 2018; Takahashi 2020). CUBIC was also applied in preclinical models to detect the vessel architecture before and after kidney injury (Hasegawa 2019). However, the main downside lies in the long clearing period for larger specimens (Kolesová 2021).

CUBIC and PACT have been intensely used to clear whole organisms by replacing the electrophoretic force with perfusion pressure, but CUBIC seems to be used more widely (Wang 2018). For example, a modified CUBIC protocol was recently presented for the mouse heart tissue clearing and imaging, which enabled the visualization of the 3D network of cardiac innervation (Nehrhoff 2016; Yokoyama 2020). However, due to EDTP, a sticky solution and copper-chelator, CUBIC does not allow a reliable evaluation of the protein loss after clearing (Susaki 2014). Indeed, it seems that CUBIC might cause a protein loss, ranging between 25 and 40% (Richardson and Lichtman 2015). On the other hand, the presence of sodium dodecyl sulfate (SDS) in the clearing solution of PACT causes heavy tissue swelling (Yang 2014).

Solvent-based clearing agents are more compatible with whole-mount immunostaining and synthetic dyes. In addition, a dehydration step is often required for organ delipidation, allowing a higher sample transparency/translucency (Dobosz 2014; Tainaka 2017; Cai 2019; Hong 2020; Huang 2019; Brenna 2020). Dobosz and coworkers used the BABB technique for visualizing breast tumors, discerning the tumor morphology, vessel architecture, and drug distribution in breast cancer from xenograft mouse models (Dobosz 2014). Moreover, using a specific fluorescently labeled antibody, they also analyzed the drug distribution within the tumor area (Dobosz 2014; Ochoa 2018). Precancerous prostate lesions have been studied by OTC-3D imaging, using 500-μm-thick tissues from radical prostatectomy specimens (Verhoef 2019). Before clearing them with BABB, the models were stained with antibodies targeting keratin 8–18 and keratin 5 to detect luminal and basal cells, respectively. Interconnecting and blind-ending saccular tubules characterize the architecture of the peripheral and transition zone of the prostate gland, and the 3D imaging was capable of revealing a new variant of prostate atrophy (Verhoef 2019).

FUnGI protocol combined with multiphoton intravital was used to image mouse mammary ducts and alveoli with a single-cell resolution (Dawson 2021). In addition, they demonstrated the compatibility of this approach with the observation of complex single-cell behavior within mammary ducts within their 3D microenvironments (Dawson 2016; Dawson 2021). Moreover, it has been shown that FUnGI is also applicable to the study of early mammary tumorigenesis and development in pregnancy for both in vivo and ex vivo models (Jamieson 2017; Linde 2018; Rios 2019). However, as described by the authors, this procedure is limited to one imaging session only, not allowing longitudinal tracking of cells over days or weeks, making longer-term processes such as mammary ductal branching challenging to analyze (Dawson et al. 2021).

For some cases, solvent-based protocols seem to overcome the most common issues faced with other approaches, such as causing changes in the tissue structure and the easy performance (Ochoa et al. 2018). For instance, lungs appear challenging to render optically transparent and 3D image due to their spongy-like consistency. Different approaches have been carried out in healthy and tumor tissues. For example, Scott and coworkers (2014) presented a BABB clearing of murine lungs and human airways for the first time, describing a complex 3D relationship between nerves, vessels, and airway architecture and detecting new patterns and connectivity of pleural innervations (Scott et al. 2014). Novel 3D studies, combining immune-staining, solvent-based OTCs (BABB or DISCO), and 3D imaging, were also performed to explore lung cancer and fibrosis, which often occurs before cancer (Cuccarese et al. 2017; Ochoa et al. 2018; von Neubeck et al. 2018). BABB was also used to image the myocardial architecture (Kolesová et al. 2021).

**Fig. 2:**
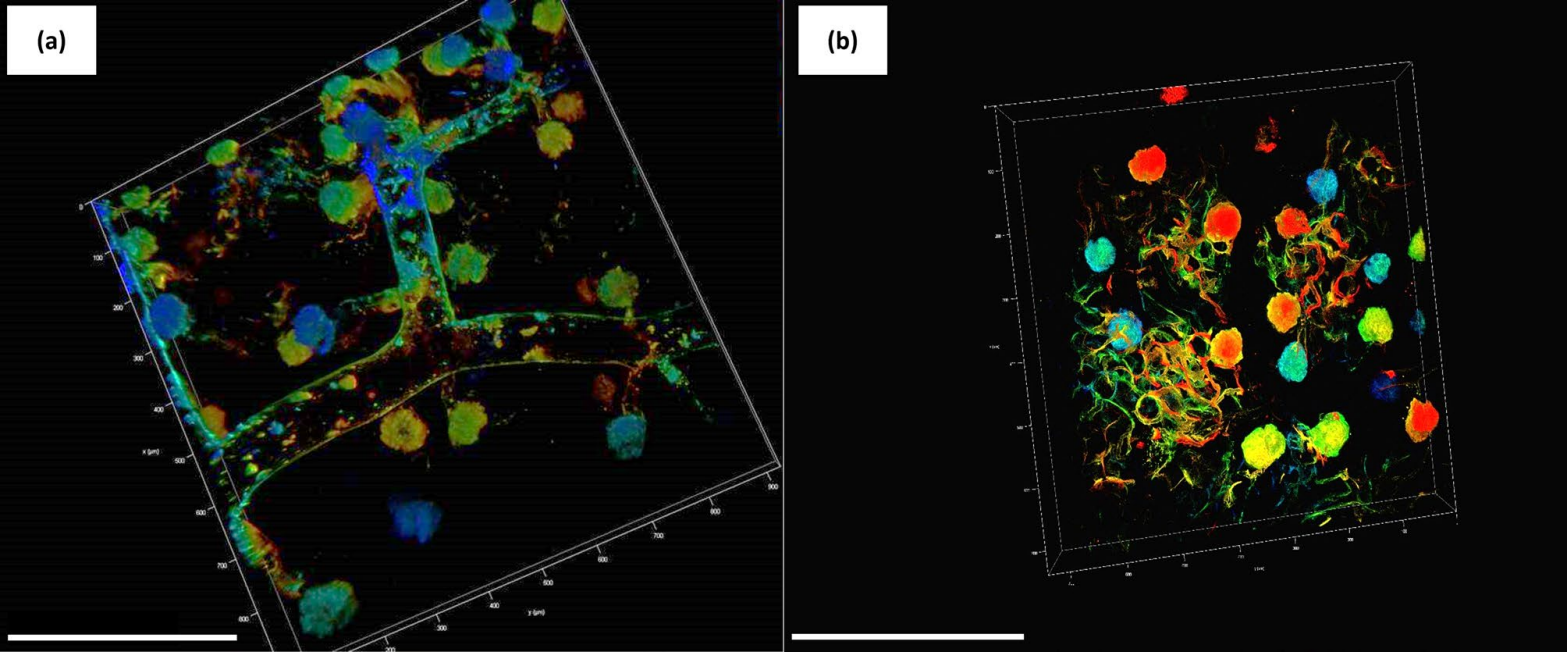
3D images of a wild-type mouse kidney, strain C57/Bl6, perfused with the cationic dye MHI148-PEI (patented according to the code WO/2018/100089), cleared by the optimized ECi protocol (see Huang et al. 2019), and imaged by CM (Leica TCS SP8, Leica Biosystem, Wetzlar, Germany), with HC PL APO 20/0.7 IMM oil CORR CS2 objective. The renal artery (**a**) and glomeruli (**b**) are shown for a total depth of 110 µm. The depth coding on the upper right part of both images displays the depth reached during the imaging. Specifically, the colored scale goes from the surface of the sample (0–50 μm, in blue–light blue), middle part (50–100 μm, in green) up to the deepest part of the region (100–150 μm, in yellow–red). Scale bars: 200 μm. The synthesis of the dye, mouse perfusion, sample harvesting, clearing, and imaging were conducted at the Zentrum für Medizinische Forschung (ZMF), Universitätsmedizin Mannheim (Germany)

However, solvent-based OTC limiting factors regard: (a) structural shrinkage due to dehydration. Moreover, with the delipidation process also being essential, structures with lipids might appear damaged and disappear; (b) the low clearing efficacy (in particular for BABB) and fluorescence preservation (Kolesová et al. 2021); (c) some pigments that increase the autofluorescence (such as hemoglobin and melatonin) are not entirely removed by the chemicals. Therefore, when studying mouse models, perfusion with PBS and PFA can optimize these limitations by reducing the autofluorescence and the heme content (Huang et al. 2019; Brenna et al. 2020); (d) some chemicals are harmful to the operator; e) the high environmental impact due to the chemicals used; and (f) the long process protocols, which can last from days to weeks. A solution could be found in optimizing the temperature for making more accessible the optical reagent into the tissues (Huang et al. 2019; Brenna et al. 2020). This is the case with ECi clearing, which Klingberg and coworkers (2017) developed and Huang (2019) then optimized. The original protocol is based on a long incubation time (≈16 h) with an increasing ethanol gradient for delipidation and a further incubation with ECi for the sample clearing. The optimization allowed shortening the incubation time by automating the whole process and modifying the temperature (from 4 °C to room temperature) (Huang et al. 2019). Figure 2a, b shows an example of the optimized ECi, where renal arteries, stained with MHI148-PEI (patented according to the code WO/2018/100089) and imaged by confocal microscopy, are depicted (Huang et al. 2019). For all the technical details, refer to Huang et al. (2019) and Brenna et al. (2020).

### Hydrogel-based OTCs in preclinical models

The hydrogel-based methods (i.e., CLARITY, PACT) are suitable for clearing many organs in mouse models (Lee et al. 2014), albeit only a few robust data describe their use in clinical applications. The presence of a polymeric matrix protects biomolecules and retains endogenous fluorescence, and the transparency rate is near 100%; the tissues present expansion after clearing. These protocols were also developed to overcome the fluorescence quenching issues and preserve the tissue structures better (Chung et al. 2013). Lipid extraction combining CLARITY with electrophoretic lipid extraction increases the rate. However, the heating (the electrophoretic CLARITY is performed at 42 °C) might damage the sample. Therefore, PACT was designed to passively clear the a hydrogel-embedded sample with a faster SDS diffusion into the specimens (Treweek et al. 2015; Yang et al. 2014; Gradinaru et al. 2018). Data from both Yang et al. and Tomer and co-workers (2014) showed a moderate tissue expansion, which could be exploited to analyze smaller structural details with higher resolution. Both the retention of fluorescence and the clearing efficacy are very high (Tomer et al. 2014). In fact, the use of hydrogels does not protect only the biomolecules from degradation, but it also efficiently retains the endogenous fluorescence (Gradinaru et al. 2018). The major downsides lie in the long time required for the sample preparation, the use of toxic chemicals for both the hydrogel (e.g., acrylamide and bisacrylamide), and the reagent for the lipid removal (SDS, as mentioned before). Some alternatives have been proposed, such as, for instance, the FxClear protocol (Choi et al. 2019), a free hydrogel electrophoretic OTC method, for rapidly removing lipids and preserving the tissue immune reactivity. By removing the acrylamide, Choi and collaborators (2019) were able to increase both the imaging resolution and the retention of the fluorescence on the mouse brain. Moreover, when acrylamide-based techniques are used, the clearing of some samples is more difficult, especially in muscular tissue (Williams et al. 2019). To overcome these limitations, another method was recently described, Myoclear (Williams et al. 2019), based on hydrogel embedding. This procedure has been established for staining and clearing neuromuscular junctions and diaphragms from healthy and diseased mice, suitable for different staining procedures and the quantification of the neuromuscular junctions (Williams et al. 2019).

In preclinical glioblastoma models, CLARITY was tested to analyze brain vasculature and tumor microenvironment, allowing a more profound knowledge of glioblastoma networks (Lagerweij et al. 2017).

A refined CLARITY method was recently established and applied in mouse hearts, allowing the visualization of cardiac fibroblasts in vivo, both in uninjured and injured samples, for characterizing and better delineating the fibroblasts in the mouse heart (Fischesser et al. 2021). This advanced protocol overcame the limitations in existing tissue-clearing protocols that have attempted to identify specific cell types in the adult or neonatal heart (Nehrhoff et al. 2016; Kolesová et al. 2016; Yokoyama et al. 2017; Wang et al. 2018). PACT was used in the first studies for mouse heart clearing, but this method consented to reaching only 80 μm of depth into the tissue (Yang et al. 2014; Tanaka et al. 2016; Fischesser et al. 2021).

Fixation for maintaining fibroblast fluorescence, a decolorization solution for reducing the autofluorescence created by the heme group within hemoglobin and myoglobin and enhancing the fluorescence from the reporter molecules, and equilibration of the tissue in refractive index matching solution (RIMS) are the steps optimized for CLARITY in this research (Fischesser et al. 2021). As a result, it could be possible to obtain images with a very high rate of tissue transparency, even in the ischemic areas, which are notoriously difficult to clear, reducing the autofluorescence produced by the fibroblast and the content of protein loss, and discriminating between healthy and injured cardiac tissues (Fischesser et al. 2021).

However, the long preparation times and toxicity of the chemicals used are still the main downsides. In addition, active CLARITY is characterized by the risk of epitope/protein loss, resulting in an incorrect structure visualization (Kolesová et al. 2016; Lagerweij et al. 2017; Chen et al. 2019; Gómez-Gaviro et al. 2020).

### Clinical applications

OTC-3D imaging might become an important tool for cancer diagnosis, as discussed previously. However, compared to animal models, such methodology might be more difficult. The two main pitfalls of this procedure are the impossibility of performing the perfusion in humans and the inaccessibility of organs. Secondly, many OTCs require immunostaining before the procedure, which is not always compatible with biopsies. Nevertheless, the new advances in tissue clearing, antibody penetration, and microscopy offer their potential to obtain detailed 3D images of cancer tissue. For example, cleared formalin-fixed paraffin-embedded biopsy samples from pancreatic, extrahepatic, and prostate tumors were successfully cleared, obtaining detailed 3D images from the cancerous areas (van Royen et al. 2016; Hong et al. 2020; Yoshizawa et al. 2020; Kolesová et al. 2021) and a visual description of a fatty invasion from a cleared human diabetic pancreas (Tang et al. 2018).

Different studies showed how solvent- and hydrogel-based protocols were used to give a more detailed comprehension of the histopathology in cancer of different human organs (Tanaka et al. 2017; Hong et al. 2020; Avilov 2021), and their potential suitability with a standard clinical workflow also been demonstrated, being compatible with formalin-fixed human specimens. In addition, ECi series is compatible with the fluorescence retainment and autofluorescence staining (Roshchina 2012; Hsueh et al. 2017; Chen et al. 2019; Brenna et al. 2020). Indeed, researchers have been reassessing the role of autofluorescence in recent times as a tool to track the inner structures, both as primary staining and in co-staining (Roshchina 2012; Brenna 2020). Autofluorescence is the primary and natural fluorescence provided by specific cellular components, such as hemoglobin, NADPH, aromatic amino acids, lipo-pigments, flavin, porphyrin, and some elements of the extracellular matrices (Rost 1992; Mori 2012; Roshchina 2012; Fred 2017). It has been demonstrated that it is possible to (i) acquire meaningful macro- and microanatomy images from different samples (e.g., mammalian glands, kidneys); (ii) discriminate between healthy and pathologic specimens by re-vitalizing old paraffin blocks, with following optical clearing and 3D imaging by confocal or multiphoton microscopy (Sabdyusheva Litschauer et al. 2020); and (iii) use the autofluorescence signal, provided by lipo-pigments or residual erythrocytes in the sample, to obtain accurate 3D spatial data and determine the shape and the position of individual cells, without the risky use of harmful chemicals for the operator and diminishing also the environmental and economic impacts (Mori et al. 2012; Layla et al. 2016). The risk in using autofluorescence is the depth reached that is lower than in other fluorescent markers. Despite OTC potentially also being clear in clinical routine, several key challenges still remain: the majority of these methods are not suitable for soft, fragile, and irregular tissue targets such as those commonly found in clinical settings, so a better optimization of these approaches is necessary; the use of specialized (and in some cases, corrosive) chemical tools represent obstacles for full adoption in clinical settings (Hsueh et al. 2017); the amount of data obtained after a 3D imaging, which ranges between gigabytes and terabytes, depends on the area analyzed, and this could be a problem both for clinicians and for patients. Moreover, all the necessary equipment to perform an OTC is highly expensive, and this could represent a strong limitation for those who cannot afford high costs for this diagnostic method.

## OTC alternatives: 3D imaging without optical clearing

Different imaging technologies can be used to obtain a 3D image at a high level of resolution, up to nanometers, which do not require cleared specimens. They could be grouped into techniques that require or do not require the volume-rendering application. Table 2 presents an overview of the most used clinical and medical imaging approaches that do not need optical clearing to obtain 3D images. The full name of the methodologies is listed in the section “Abbreviations.”

**Table 2:** Overview of methodologies involving 3D imaging without optical clearing and mostly used in the medical field

Technique	Medical Imaging Sector	Reference(s)
CT scan	Pneumoencephalography; bronchography; upper gastrointestinal and lower series; cholangiography; mammography; angiography; venography	Leeds and Kieffer et al. (2000); Ronald et al. (2005); Tonelli et al. (2011); Bates et al. (2012); Mori et al. (2012); Unett et al. (2013); Murphy et al. (2014); UK (2016)
MRI	Brain; neurography; cardiac (perfusion); angiography; cholangiopancreatography;	Villringer (1988); Howe (1992); Prasad et al. (2001); Campeau and Huston et al. (2012); von Knobelsdorff-Brenkenhoff et al. (2017)
Ultrasound	Ecocardiography; gynecologic; obstetric; echoencephalography; abdominal; ultrasonography carotid	Caspi et al. (2003); Whitworth et al. (2015); Cleve et al. (2018); Saxena et al. (2019)
Tomography Array tomography CT ECT Magnetic induction tomography MicroCT	Brain connectome; myocardial imaging; cancer monitoring	Koffie et al. (2009); Strauss and Bailey et al. (2009); Soiza-Reilly and Commons et al. (2011); de Calignon et al. (2012); Flottmann et al. (2012); Nanguneri et al. (2012); Kopeikina; et al. (2013); Whitworth et al. (2015)

The volume rendering-based techniques acquire images of 2D single slices that are then reconstructed into a 3D volume using specific imaging software programs. The images are usually acquired in a regular pattern (e.g., one slice every millimeter) with a standard number of image pixels. Examples of these techniques are computed tomography (CT) scan, magnetic resonance imaging (MRI), and X-ray microtomography (MicroCT).

Scanning transmission electron microscopy (STEM) does not include volume rendering image post-processing.

In STEM, the electron beam hits the sample in focus, and each pixel of the generated image contains information corresponding to the precise location of the sample with sub-ångström resolution (Crewe and Wall 1970). In medical sciences, STEM has been used to characterize the morphology of amyloid fibrils (Petkova et al. 2002; Diaz-Avalos et al. 2005).

During recent decades, STEM was combined with the serial block face approach (SBF-SEM) in different fields of life science research (Lippens et al. 2019). SBF-SEM is a relatively new technique, allowing the acquisition of serially sectioned, imaged, and digitally aligned ultrastructural data (Cocks et al. 2018). The main advantage of this technology lies in covering a range of volumes, from monolayers to multiple tissue layers, in all three dimensions. SBF-SEM was initially used in neuroscience and then expanded to other biological specimens, such as animal tissues, unicellular organisms, and plants (Borrett and Hughes 2016). Recently, SBF-SEM has been applied in murine models to explore the hepatic microarchitecture, allowing the assessment of large-volume morphometric data on parenchymal cells, sinusoids, and bile canaliculi, and it was also exploited to investigate the human placental microvasculature, revealing new intercellular connections (Shami 2016).

A wealth of information can be obtained from the resulting image stacks, albeit the computational analysis of the huge data sets produced still represents a new challenge for the researchers (Cocks et al. 2018). One approach is to reconstruct structures and features of interest in 3D. However, the software programs might be overwhelming, time-consuming, and not intuitive for image analysis. Moreover, the alignment of the single slices could also be prone to error, and the sample preparation process for SBF-SEM is very long and consists of many elaborate steps (Lippens et al. 2019). Lastly, this 3D reconstruction covers only the outer face of the samples without investigating the inner structures and limiting the analysis only to the superficial features. Because of these limiting factors, only a limited number of published articles provide sufficient detail on this type of reconstruction (Cocks et al. 2018).

In its several variants, tomography is considered the progenitor of the volume rendering-based methodologies. MRI and CT are widely used in the medical field, especially for cancer monitoring, and they are both used to capture images within the human body. The main difference between them is that MRI uses radio waves and CT X-rays. For a detailed technical description of this imaging, refer to Kemmerer (1998) and Grover et al. (2015).

MRI was shown to be superior in regards to the image’s detail. MRI can perform in vivo imaging in two ways: single-voxel spectroscopy and chemical shift imaging. Single-voxel spectroscopy defines a voxel of interest within organs using gradients. The size of the voxel is predefined by the user and is the only source of the signal. Therefore, the number of signal averages acquired may be increased, requiring increased scanning time to improve the signal-to-noise ratio in smaller voxels. Chemical shift imaging (CSI) acquires spectra from a matrix of voxels, although it is usually done in one plane, instead of the three directions, hence the name 2D-single slice CSI. The advantage of single-voxel spectroscopy is in increasing the signal-to-noise ratio, whereas CSI allows wider anatomical coverage (Grover et al. 2015).

A CT scanner uses a motorized X-ray source, rotating around the circular opening of a donut-shaped structure called a gantry. During a CT scan, ionizing radiation (X-rays) coupled with an electronic detector records a density pattern and creates an image resulting from slices from the tissue (Caldemeyer and Buckwalter 1999). The X-ay beam rotates around the object within the scanner. Multiple X-ay projections pass through the object, and the object’s internal structure is reconstructed from the multiple projections of that object (Caldemeyer and Buckwalter 1999). The tissue thickness represented in each image slice usually ranges from 1 to 10 mm (Science Education 2013, https://www.nibib.nih.gov/science-education/science-topics/computed-tomography-ct). When a full slice is completed, the image is stored, and the X-ay scanning process is then repeated to produce another image slice (Science Education), repeating the process until the chosen number of slices is collected (Science Education). It is possible to acquire 3D images of the skeleton, organs, tissues, and any abnormalities. This method has many advantages, including rotating the 3D image in space or viewing slices in succession, making it easier to find the exact place where a problem may be located. Other differences between MRI and CT scans include their risks and benefits. CT scan risks consist of a very small dose of radiation and a potential side reaction to the use of the markers, whereas the main MRI risk is related to possible reactions to metals due to magnets (Science Education).

Both MRI and CT scans can view internal body structures. However, CT scanning is faster and provides images of tissues, organs, and skeletal structures. On the other hand, MRI is highly adept at capturing images to determine abnormal tissues within the body. In addition, MRI provides more high-resolution images and, therefore, with more details.

The post-process imaging for both CT scanning and MRI involves using specific voxel-based morphometry algorithms (Dousset 1992; Rugg-Gunn et al. 2006; Flohr and Ohnesorge 2007). Post-processing methods represent an important improvement to conventional visual analysis but need to be interpreted with expertise to be apprehended as a complementary tool within the multimodal evaluation of the diseases (Martin 2015). Array tomography, developed by Micheva and Smith (2007), combines and extends the features of optical fluorescence and scanning electron microscopy, and it was initially designed to study synapses in the rodent brain (Micheva and Smith 2007). This method is based on embedding a tissue specimen in acrylic resin and cutting it into a series of very thin sections (50–200 nm) and gluing them to glass slides. The resulting array is labeled with fluorescent markers and imaged to generate ultra-high-resolution volumetric images. The array can be repeatedly eluted, restained, and fluorescently imaged, and finally, it can also be stained with heavy metals and imaged with a scanning electron microscope (Micheva and Smith 2007). In mouse models, it has been used to characterize the synaptic protein composition in healthy brains (Micheva and Smith 2007; Soiza-Reilly and Commons 2011; Nanguneri 2012) and to demonstrate synaptic loss and proteins involved in synaptic degeneration in Alzheimer’s disease models (Micheva and Smith 2007; Koffie 2009; de Calignon 2012; Kopeikina 2013).

Kai and coworkers (2013) used this methodology on postmortem human brains to investigate synapse degeneration in Alzheimer’s disease, the contributions of specific apolipoproteins in Alzheimer’s disease risk, and the mitochondrial size and distribution in neurons of a tauopathy mouse model and human Alzheimer patients. In addition, array tomography is suitable for examining many other small biological structures’ morphology, localization, and protein composition. For example, it was exploited to investigate the microstructural changes in blood vessel walls during abdominal aortic aneurysms in mice (Saatchi 2012), or the collagenous matrix of the human optic nerve head (Winkler 2010). Furthermore, this approach guarantees images with a better spatial resolution than confocal microscopy, although it is limited only to fixed specimens; it is also not suitable for in vivo imaging (Micheva and Smith 2007), and all the procedure is cumbersome and highly time-consuming.

In general, besides the great advantages and benefits of these technologies in the medical and diagnosis fields, there are still some downsides. First, these procedures might give inexact results. They represent a compromise between the accuracy and computation time required, leading to producing artifacts (errors in the reconstruction) at a higher computing cost (Herman 2009). Moreover, all these approaches do not image the inner structures but only the surface of the samples. In modern society, where several diseases have been rising, it would be relevant to know and better decipher the structural changes within the organ in order to define the primary cause of the disease and not only the consequences, as, for instance, the abnormal tissue, which is generated from previous steps.

## Conclusions and future perspectives

The increasing need to discover anatomic details leads researchers to develop and design more accurate technologies to be included in clinical applications. 3D imaging is deeply rooted in routine and cancer fields, although only superficial anatomic features can be imaged so far.

Over the recent decades, the roles of OTC and 3D fluorescent imaging in the clinical field have been gaining importance, given the possibility to follow the development of the pathology and establish new medical treatments. Furthermore, with the advent of organoids and spheroids, it is possible to think about a new and modern 3D biology, both in vitro and in vivo, with preclinical and clinical studies.

The high-resolution 3D imaging of protein structures within cleared tumors appears necessary to understand the development of a disease, especially in oncology, considering the high variability of the tumor environment.

With this review, we wanted to assess the state of the art of 3D imaging in clinical applications. OTC-3D imaging is still far from being included in routine and cancer diagnosis and monitoring, but this approach might facilitate and increase new knowledge in tumor biology by facilitating the discovery of new pharmaceutical targets and quantifying the drug penetration. Moreover, the 3D study of the penetration and distribution rate and its interaction in the target region may provide advanced information about treatment responses. Therefore, it is necessary to better understand the drug behavior in the whole human body to further develop personalized medicine. Moreover, researchers should be encouraged to design more accurate OTCs, which could be used in patients, to establish a more tailored medicine.

## Data Availability

Not applicable.

## Abbreviations

2D: Two-dimensional
3D: Three-dimensional
BABB: Benzyl alcohol/benzyl benzoate
CLARITY: Clear lipid-exchange acrylamide hybridized rigid imaging in situ hybridization-compatible tissue hydrogel
CSI: Chemical shift imaging
CT: Computed tomography scanning
CUBIC: Clear, unobstructed brain/body imaging cocktails and computational analysis
DISCO: Dimensional imaging of solvent-cleared organs
Eci: Ethyl cinnamate
ExM: Expansion microscopy
FRUIT: Fructose and urea cocktail
FUnGI: Fructose, urea and glycerol for imaging
GFP: Green fluorescent protein
GBM: Glioblastoma multiforme
MicroCT scan: X-ray microtomography
MRI: Magnetic resonance imaging
OTC: Optical tissue clearing
OTC-3D imaging: Optical tissue clearing combined with 3D imaging
PACT: Passive clearing tissue
PEGASOS: Polyethylene glycol (PEG)-associated solvent system
SBF-SEM: Serial block face-scanning electron microscopy
seeDB: See deep brain
STEM: Scanning transmission electron microscopy
